# Endo-cannabinoids system and the toxicity of cannabinoids with a biotechnological approach

**DOI:** 10.17179/excli2017-257

**Published:** 2017-05-15

**Authors:** Kamal Niaz, Fazlullah Khan, Faheem Maqbool, Saeideh Momtaz, Fatima Ismail Hassan, Navid Nobakht-Haghighi, Mahban Rahimifard, Mohammad Abdollahi

**Affiliations:** 1 International Campus, Tehran University of Medical Sciences (IC-TUMS), Tehran, Iran; 2 Toxicology and Diseases Group, Pharmaceutical Sciences Research Center, Tehran University of Medical Sciences, Tehran, Iran; 3 Department of Toxicology and Pharmacology, Faculty of Pharmacy, Tehran University of Medical Sciences, Tehran, Iran; 4 Medicinal Plants Research Center, Institute of Medicinal Plants, ACECR, Karaj, Iran; 5 Faculty of Pharmacy, Eastern Mediterranean University, Famagusta, North Cyprus Mersin 10, Turkey

**Keywords:** synthetic cannabinoids, acute, chronic, toxicity, biotechnology

## Abstract

Cannabinoids have shown diverse and critical effects on the body systems, which alter the physiological functions. Synthetic cannabinoids are comparatively innovative misuse drugs with respect to their nature of synthesis. Synthetic cannabinoids therapy in healthy, chain smokers, and alcoholic individuals cause damage to the immune and nervous system, eventually leading to intoxication throughout the body. Relevant studies were retrieved using major electronic databases such as PubMed, EMBASE, Medline, Scopus, and Google Scholar. The extensive use of *Cannabis Sativa *L. (*C. Sativa*) and its derivatives/analogues such as the nonpsychoactive dimethyl heptyl homolog (CBG-DMH), and tetrahydrocannabivarin (THCV) amongst juveniles and adults have been enhanced in recent years. Cannabinoids play a crucial role in the induction of respiratory, reproductive, immune and carcinogenic effects; however, potential data about mutagenic and developmental effects are still insufficient. The possible toxicity associated with the prolong use of cannabinoids acts as a tumor promoter in animal models and humans. Particular synthetic cannabinoids and analogues have low affinity for CB1 or CB2 receptors, while some synthetic members like Δ9-THC have high affinity towards these receptors. Cannabinoids and their derivatives have a direct or indirect association with acute and long-term toxicity. To reduce/attenuate cannabinoids toxicity, pharmaceutical biotechnology and cloning methods have opened a new window to develop cannabinoids encoding the gene tetrahydrocannabinolic acid (THCA) synthase. Plant revolution and regeneration hindered genetic engineering in *C. Sativa. *The genetic culture suspension of *C. Sativa* can be transmuted by the use of *Agrobacterium tumefaciens *to overcome its toxicity. The main aim of the present review was to collect evidence of the endo-cannabinoid system (ECS), cannabinoids toxicity, and the potential biotechnological approach of cannabinoids synthesis.

## Introduction

Humans and animals naturally synthesize and exert a group of chemical compounds (ligands) named endo-cannabinoids that activate their receptors located throughout the central and peripheral nervous systems. The ECS (Endo-Cannabinoids System) consists of cannabinoid receptors and endo-cannabinoids compounds that secrete neurotransmitters (NTs) throughout the body and especially in the brain. In the 1990s, the first and second cannabinoid receptors, CB1 and CB2 were cloned and classified as the member of the family of G protein-coupled receptors. The CB1 is found abundantly in brain neurons, while CB2 is found primarily in cells of the peripheral immune system. Maintenance of the homeostatic and physiologic functions of the body is considered as the major tasks of ECS (Fine and Rosenfeld, 2013[53]). In addition, phytocannabinoids (exogenous plant-derived cannabinoids) are naturally produced ligands in *C. Sativa* and some other plants, of which synthetic cannabinoids are synthesized from the ancient time (Pacher et al., 2006[114]). The most recognized phytocannabinoids are known as tetra-hydrocannabinol (THC); the major psychoactive compound in cannabis (Lambert and Fowler, 2005[89]; Santos et al., 2015[137]). Other main phytocannabinoids ingredients include: cannabidiol (CBD) and cannabinol (CBN). Almost 85 various cannabinoids have been identified and isolated from *C. Sativa* showing different health effects (El-Alfy et al., 2010[50]). 

Few decades ago, it has been understood that ECS performs several normal body functions. The potential medical activities of the ECS have been explored in the last years. Cannabinoids have numerous biological and functional properties, which modulate ECS with agonists and antagonists with novel therapeutic purposes towards various disorders. For instance, 'anandamide' has the potential to increase food intake in rats (Costa et al., 1999[34]) and raise the weight in cancer and human immunodeficiency virus (HIV+) patients; while 'SR-141716 A' as an antagonist prohibits food intake (Arnone et al., 1997[8]; Colombo et al., 1998[32]; Simiand et al., 1998[143]) and acts as a vital anti-obesity remedy which functions on CB1 receptors in the hypothalamus (Berry and Mechoulam, 2002[17]).

Within the body, endo-cannabinoids act as ligands intended for cannabinoid receptors, thus play as neuromodulators role in the brain. Ligands are small molecules able to dock onto the binding site of the proteins, therefore accomplish their ability to regulate the receptors' function and its downstream biological pathways. The basic building block unit of endo-cannabinoids are polyunsaturated fatty acids, hence the only difference in the chemical composition from phytocannabinoid of the cannabis plant. The well-known endo-cannabinoid compounds include; anandamide, 2-arachidonoylglycerol, 2-arachidonylglyceryl ether (noladin ether), O-arachidonoyl-ethanolamine (virodhamine) and N-arachidonoyl-dopamine (NADA) (Grotenhermen, 2004[62]). Anandamide and NADA are not only responsible for binding to cannabinoid receptors, but also upregulate the ability of capsaicin, an essential part of hot chili peppers, to modulate vanilloids (TRPV1) receptors.

Several studies have been revealed that cannabinoids stimulate the ECS. During the painful situation, the endo-cannabinoids are elevated in the periaqueductal gray of the brain following painful stimuli. The activation of cannabinoid receptors, resulting in nerve damage was described in the animal study of long-lasting neuropathic pain and intestinal inflammation. In such conditions, cannabinoid agonist effectiveness was enhanced (Grotenhermen, 2006[61]).

The ECS along with biological and physiological properties of cannabinoids has been extensively studied in the past decades. It is noteworthy that cannabinoids have immune modulatory effects and their possible role in autoimmune disease and inflammatory therapy has been investigated. However, the main aim of this review was to collect evidence of ECS and toxicity of cannabinoids with the potential biotechnological approach of cannabinoids.

## Methods

### Search strategy

**Figure 1:**
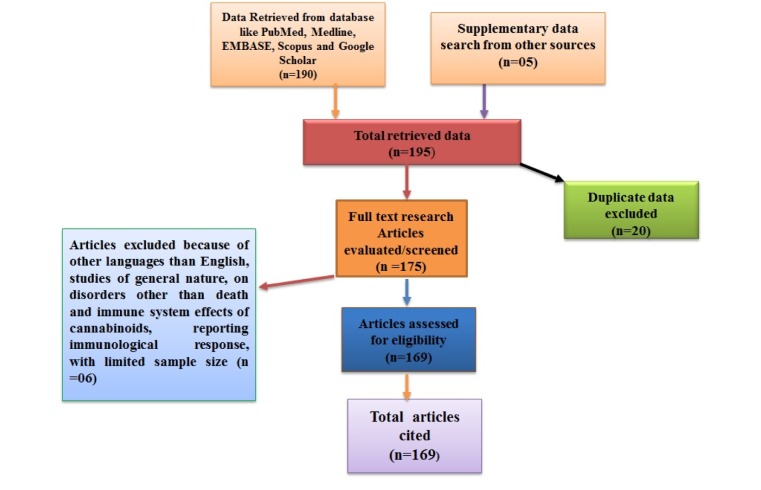
Flow diagram of included studies. The flow chart depicts the number of citation and resource materials that have been screened, excluded and/or included in the review.

The studies comprised in present review have been retrieved from the PubMed database of the National Library of Medicine, Scopus, EMBASE, Medline and Google Scholar by setting limits for papers published mostly from 1990 onwards, however few before, using the keywords “endo-cannabinoids system”, “biotechnology and cannabinoid”, “toxicity of cannabinoids”, “acute toxicity of cannabinoids”, “moderate effects of cannabinoids”, “chronic effects of cannabinoids”, and “cannabinoids and immune system”. This bibliographic search retrieved 250 studies (Figure 1(Fig. 1)).

### Exclusion and inclusion criteria

Criteria for exclusion were the reports in languages other than English, studies for which abstract was not present, reports concerning to the association of cannabinoids with studies other than death and the immune system. Most studies, which investigated the relation of toxicity of cannabinoids on the non-cannabinoid system, were also excluded. The studies, which focus on single or limited cases having adverse effects without showing a clear role of cannabinoids in the toxicological pathogenesis, were also excluded. Eventually, a total number of 195 reports indexed in Google Scholar and/or PubMed were found to gratify the criteria of inclusion. Various studies not indexed in PubMed were obtained by manual searching in Google Scholar, and such reports which satisfied the criteria for inclusion were further retrieved. Therefore, the total number of studies (n) included in this review reached 169 (Figure 1(Fig. 1)).

## The ECS

In short, cannabinoid receptors, their endogenous ligands and the enzymes that synthesize and degrade endo-cannabinoids construct the ECS (Mackie, 2008[97]). The following sections will focus on the cannabinoid receptors such as CB1 and CB2 along with other non-CB receptors, which exert their effects by regulating NTs and cytokine release.

### Cannabinoids receptor agonists

**Figure 2:**
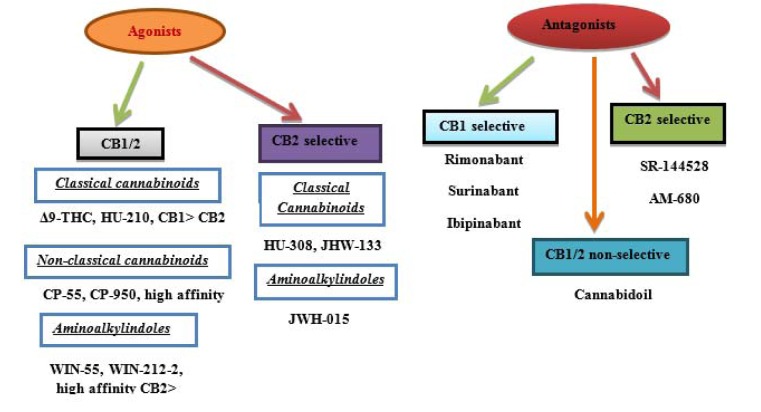
Agonists and antagonists of cannabinoids receptors. Both CB1 and CB2 receptors of agonists and antagonists classes have been illustrated. Synthetic derivatives such as HU-210, CP 55/950 and HU-308 are the most efficient compounds used for pharmacological purpose.

Today, several compounds have been prepared capable of acting as agonists against both cannabinoid receptors. On the basis of heterogeneous chemical structure, cannabinoid receptor agonists are categorized into four types of groups such as: classical, non-classical, eicosanoid and aminoalkylindole compounds as shown in the Figure 2(Fig. 2) (Howlett et al., 2002[75]; Pertwee, 2005[124]). Briefly, the classical group involves phytocannabinoid (Δ9-THC, cannabinol) and their synthetic derivatives. The non-classical group consists of bicyclic and tricyclic analogues of Δ9-THC that lacks a pyran ring such as P55940, HU-308, CP47497, and CP55244. The endo-cannabinoids produced by our body cells is categorized in the eicosanoid group. These endo-cannabinoids stand for arachidonoylethanolamide, *O*-arachidonoylethanolamine, 2-arachidonoyl glycerol, 2-arachidonyl glyceryl, and numerous other synthetic analogues of anandamide. Aminoalkylindole comprises of WIN5512. 

The cannabinoids mostly act on two important receptors of the ECS, CB1 and CB2 receptors. Each cannabinoid has its own affinity for the specific receptors (Howlett et al., 2002[75]; Pertwee, 2005[124]). For example, Δ9-THC has a high affinity towards CB1 receptor, while cannabinol is an agonist without marked CB1/CB2 selectivity (Figure 2(Fig. 2)). 

### Cannabinoid receptors

#### Cannabinoid receptors CB1 and CB2

Generally, CB1 receptors are mostly present in several brain regions; more precisely exist in the basal ganglia, in the limbic system comprising the hippocampus and to a lesser extent in other parts of the body (Pacher et al., 2006[114]). These receptors mediate many of the psychoactive effects of cannabinoids (Mackie, 2008[97]). CB1 receptors have also been observed in the cerebellum and in both male and female reproductive systems. CB2 receptors have shown more restricted distribution, being found in the immune system and in a few neurons, with extremely high quantity in the spleen. They are also expressed by microglia in the human cerebellum (Núñez et al., 2004[110]; Pacher and Mechoulam, 2011[115]). Animal studies have shown that CB2 receptors might be responsible for their anti-inflammatory and other therapeutic activities (Pacher and Mechoulam, 2011[115]). Theoretical overview, physiology, functions and applications of these receptors have been discussed extensively by researchers.

**Figure 3:**
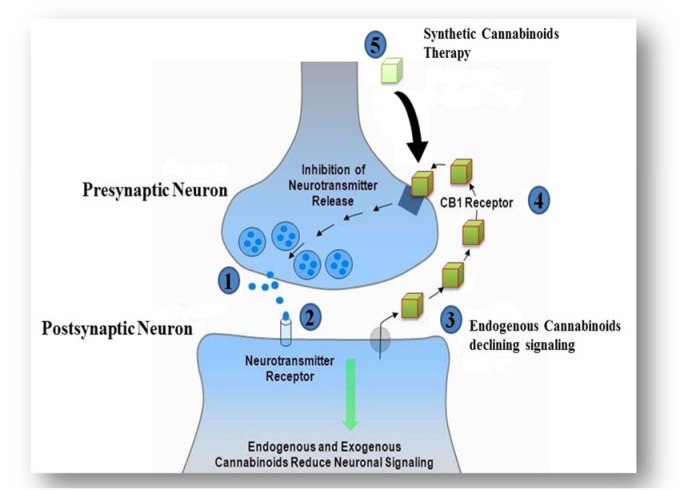
Mechanism of action of the cannabinoids at pre- and postsynaptic terminal. 1). NT from presynaptic neuron triggers the postsynaptic neuron. 2). Stimulated postsynaptic neuron releases endo-cannabinoids. 3). Endogenous CB1 ligand disseminates back to and binds to the presynaptic CB1 receptor. 4). CB1 receptor stimulates a G-protein, leading to inhibition of neurotransmitter release. 5). Synthetic cannabinoids are thought to activate CB1 receptors directly, imitating the effects of endo-cannabinoids.

Synthetic cannabinoids acting on CB1 and CB2 receptors as agonists exhibit their therapeutic effects such as anti-inflammatory, bronchodilation, anti-allergic effects, neuroprotection, antineoplastic, appetite stimulator, pleasure sensation, mood, memory, immune system stimulator, anti-nausea properties, and pain killer, while restraining the psychoactive properties (Grotenhermen, 2004[62]). Synthetic cannabinoids inhibit the excess NTs at the junction of pre- and post-synaptic neurons, which ultimately mimic the effects of endo-cannabinoids (Figure 3(Fig. 3)). The therapeutic effects of cannabinoids are also attributed to generating extensive toxicity in different systems (Gurney et al., 2014[67]). During the past decade, it was discovered that synthetic cannabinoids have a higher affinity towards CB2 receptors. Toxicology laboratories around the globe have made intensive efforts to keep up with the rate, at which cannabinoids are designed and marketed (Zawilska and Wojcieszak, 2014[165]). However, for the assessment of pharmacokinetics limitations, inadequate data of controlled studies do exist (Kronstrand et al., 2013[86]). The association between the influence and the concentration of cannabinoids is not clearly well-defined.

#### Mechanism of action of CB1 and CB2 receptors

The binding of heterotrimeric G_i/o_ proteins to CB1 and CB2 receptors produce various effects. The activation of G alpha i/o proteins triggers CB1 receptors to exhibit their effects. The inhibition of adenylate cyclase enzyme occurs due to the coupling of CB1 to its agonists. Similarly, increase in the level of mitogen-activated protein kinase (MAPK) causes lowering of intracellular cAMP level due to binding of CB1 and its ligands. In certain situations, due to the activation of CB1 receptors attached to G_s_ proteins stimulate adenylate cyclase-cAMP (Di Marzo, 2008[44]; Pertwee, 2006[122]). CB1 and CB2 receptors also are involved in a variety of ion channels in the cell membrane, which are completely penetrating correct the calcium and potassium channels. The binding of cAMP-dependent intact of receptors with molecules such as c-Jun, c-fos, p38, N-terminal kinase (JNK), extracellular signal controlled kinase (ERK), Rf-1, protein kinase-C (PKC) and protein kinase p (PKA), so these calcium and potassium channels are stimulated (Pagotto et al., 2006[117]). In the case of CB1, initiation can lead to lessening of Ca^2+^ ion access into the cell, without the presence of cAMP, which is necessary for the NT release. As a whole, they would affect a decrease in the release of NTs. Therefore, in dose-response relationship manner, CB1 receptor is a pre-synaptic junction that moderates the release of NTs (Howlett, 2005[74]). 

CB1 and CB2 cannabinoid receptors also have the ability to control phosphorylation and initiate many members of MAPKs, such as p38 MAPKs, c-Jun, extracellular signal-regulated kinase-1 and -2 (ERK1/2). Besides, this MAPK also regulates gene expression associated with cell motility, proliferation, apoptosis and glucose metabolism (Howlett, 2005[74]). The involvement of endogenous, exogenous and synthetic agonists with CB1 and CB2 receptors produces their desirable effects. After the anticipated effects, agonist molecules are rapidly neutralized by entry into the cells and are metabolized. The metabolic process of hydrolysis of 2-AG by monoglyceride lipase or enzymatic hydrolysis with the help of fatty acid amide hydrolase enzyme (FAAH) metabolizes anandamide (Di Marzo, 1998[43]; Dinh et al., 2002[46]; Giuffrida et al., 2001[56]). 

#### Cannabinoid receptors and their regulations

CB1 receptors are located in particular non-neuronal cells and in all central and peripheral neurons (Howlett et al., 2002[75]; Pertwee, 1997[125], 2005[124]). In the central nervous system (CNS), the dissemination patterns of the CB1 receptors are heterogeneous and linked to their function. CB1 receptors are abundantly present in the cerebellum, entopeduncular nucleus, globus pallidus, substantia nigra pars reticulate, caudate-putamen, hippocampus and cerebral cortex along with some parts of the spinal cord and other areas of the brain. They are involved or modulate in pain sensation due to stimulation of the nerve cells. Different studies suggested that the presence of CB1 receptor agonist in CNS triggered to alter the ability of perception, memory as it regulates motor function and to initiate anti-nociception (Iversen, 2003[78]; Pertwee, 1997[125], 2005[124]; Pertwee et al., 2000[126]). The CB1 receptors located in the central and peripheral nerve ending control the release of inhibitory and excitatory NTs activation (Howlett, 2005[74]; Pertwee, 2005[124]). CB2 receptors present in the immune cells are responsible for immunomodulation (Howlett et al., 2002[75]; Pertwee, 1997[125]). Both CB1 and CB2 receptors regulate each other activities to release chemical messengers in the appropriate level. By the interaction of cannabinoids, CB1 receptors release NTs at the CNS and control their release, while CB2 regulate the release of inflammatory cytokines, modulating the immune system (Marsicano et al., 2002[100]). 

### Other cannabinoid non-CB1 and non-CB2 receptors of the ECS

#### Vanilloid receptors

There is a non-CB receptor, which has the cannabinoids' conjugation capacity known as capsaicin or TRVP-1 receptor. Capsaicin receptors are mainly present in the nociceptive neurons of peripheral nervous system, however, they have been found in many other tissues involving the CNS. Capsaicin is mostly involved in the spreading and regulation of neuron pain through perivascular and primary afferent neurons (De Petrocellis and Di Marzo, 2009[39]; Devane et al., 1992[41]; Di Marzo and Petrosino, 2007[45]). It has been illustrated the conjunction of endogenous cannabinoid anandamide with capsaicin receptor leads to the release of substance-P and calcitonin gene-related-P (CGRP), which exerts a local vasodilation, allogeneic and pro-inflammatory effects along with advantageous actions like cardio-protection and anti-hypertensive properties (Ahluwalia et al., 2003[1]; Hwang et al., 2000[77]; O'Sullivan et al., 2004[112]; Price et al., 2004[129]; Shin et al., 2002[141]; Tognetto et al., 2001[151]; Zygmunt et al., 1999[169]). 

#### Non-CB1, non-CB2 and non-vanilloid receptors

Few studies have revealed that various biological activities of the cannabinoids are difficult to be reversed by CB1 and CB2 antagonists. To achieve this goal, many other receptor pathways such as G-protein receptor-55 (GPR-55), nicotine, adenosine A-2-A and peroxisome proliferator-activated receptors (PPARs), have been identified for cannabinoids signalling transduction (Klein, 2005[81]; Lazzerini et al., 2012[90]). 

#### Allosteric location of cannabinoids

Besides the aforementioned receptors, there are several allosteric sites for anandamide and other cannabinoids on the numerous non-cannabinoid receptors (Pertwee, 2003[123], 2004[121], 2005[124]). These allosteric sites include M1/M4 muscarinic receptors, α1-adrenoceptors, 5-HT3, 5-HT2, α-amino-3-hydroxy-5-methyl-4-isoxazolepropionic acid (AMPA), GLUA-1 and GLUA-3 glutamate receptors (Akinshola et al., 1999[2][3]; Barann et al., 2002[11]; Cheer et al., 1999[28]; Christopoulos and Wilson, 2001[30]; Fan, 1995[51]; Godlewski et al., 2003[57]; Oz et al., 2002[113]). However, there is no evidence of the biological significances of allosteric sites on M1/M4 receptors by anandamide and methanandamide or SR141716A and 5-HT2 receptors by HU-210 (Pertwee, 2005[124]).

### Effects of cannabinoids 

There are various effects of cannabinoids on the body systems, which includes muscle relaxation, anti-inflammation, anti-allergic, sedative, neuroprotective, anti-emesis and antineoplastic properties (Grotenhermen, 2004[62]). However, the next section would only discuss the interaction of cannabinoids with CNS. 

### Cannabinoids and CNS

The cannabinoids also show their potential effects on the CNS interrelating with various NTs and neuromodulators such as histamine, serotonin, glutamate, norepinephrine, prostaglandins, opioid peptides, acetylcholine, dopamine and gamma-amino butyric acid (GABA) (Baker et al., 2003[10]; Dewey, 1986[42]; Grotenhermen, 2004[62]; Pertwee, 1992[120]). Some of the biological activities and beneficial effects of THC can be elucidated by these correlations. For example, tachycardia and hypo-salivation with dry mouth are facilitated by the effects of THC on the release and turnover of acetylcholine (Domino, 1999[47]; Mattes et al., 1994[101]). Serotonin interacts with cannabinoids having anti-emetic properties (Fan, 1995[51]). The interactions of cannabinoids with dopamine, glutamate, and GABAergic transmitter systems are attributed to spasmodic conditions (Grotenhermen and Russo, 2013[64]). The inhibition of surplus glutamate production, prohibition of calcium influx into the cells and antioxidant properties of neuroprotective cannabinoids were detected in animal models, which lessen the level of oxygen radicals and the modulation of vascular tone (Grundy, 2002[65]; Grotenhermen and Russo, 2013[64]). In addition, cannabinoids are also studied for stroke and brain damages.

### Cannabinoids' analogues

CBG-DMH, an analogue of cannabinoids, is showing hypotensive and vascular relaxant properties (Maor et al., 2005[98]). The CB1/2, vanilloid receptor antagonists and nitric oxide synthase do not prevent vascular relaxation induced in the abdominal aorta of rat by CBG-DMH, due to pertussis toxin sensitivity. CBG-DMH reduces nitric oxide production and tumor necrosis factor (TNF) in murine macrophages. However, the effect of hypotension is unclear; it may be correlated with an abnormal cannabinoid, a CBD isomer, which inhibits the effect of these compounds (Vanessa Ho and Hiley, 2003[156]). Furthermore, CBD has not the capability to prevent mechanism of hypotension induced by THC, while hypotension may follow some new mechanisms. 

Another plant origin propyl analogue of THC is THCV, which is an antagonist of anandaminde and WIN-55212. THCV plays its role on the basis of the selectivity, which prevents the effect of both agonists in the vas deferens than brain membranes (Ashton, 2001[9]; Begg et al., 2005[14]). The influence of THCV is proven to be more potent for antagonizing the effect of WIN and anandamide on electrically induced contractions of the vas deferens than provoking the inhibition initiated by THC (Ashton, 2001[9]).

At 3-1000 nanomol (nM), THCV did not prevent electrically induced contractions of mouse isolated vas deferens; though, the amount of THCV in this range formed dextral shifts in the log concentration-response curves of WIN and anandamide for electrically evoked contractions. These changes were not convoyed by a reduction in the maximal effect of any agonist. Nevertheless, at 3 millimolar (mM), THCV did lessen the contractile reaction of the vas deferens in a CB1 receptor antagonist (SR141716)-independent way. Moreover, THCV looks like SR141716 antagonist, which at high quantities also cooperates with non-CB1 targets (Mechoulam, 2005[103]).

It has been established that very little dose of anandamide (0.0001-0.1 mg/kg), which had negligible effects when administered alone, partially or fully inhibited THC-induced effects (Fride et al., 1995[55]). It has been shown (Bayewitch et al., 1996[13]) that THC antagonizes the agonist-induced inhibition of adenylyl cyclase-mediated by the CB2 receptor and determined that THC constitutes a weak antagonist for this receptor under the circumstances of their experiments.

### Medical application of cannabinoids and their effects on immune function

Over the years, various studies have been conducted concerning cannabinoids, their roles in ECS and possible therapeutic actions. It has been proved that cannabinoids propose several physiological properties and their multiple agonists and antagonists exhibit valuable potentials in various diseases. The therapeutic footprints of cannabinoids have been traced in cancers, diabetes, rheumatoid arthritis, multiple sclerosis, glaucoma, allergic asthma, dystonia, spinal cord injuries, analgesia, Tourette's syndrome in humans, nausea and epilepsy (Amar, 2006[4]; Grotenhermen and Müller-Vahl, 2012[63]). Cannabis-based medications possess their effects via the activation of cannabinoid receptors. The conduction of various controlled clinical trials has led to the approval of several cannabis-based medicines (dronabinol, nabilone and a cannabis extract [THC: CBD=1:1]) in several countries (Grotenhermen and Müller-Vahl, 2012[63]). Beside cannabis, some other herbs such as *Ephedra sinica*, *Cissus quadrangularis*, *Momordica charantia* and *Zingiber officinal* also act on cannabinoid receptors (Hasani-Ranjbar et al., 2009[71]). 

The immune system is a complex set-up of many biological structures and functions such as cells, cytokines, hormones, tissues and physiological processes that protects the body against diseases. The presence of cannabinoid receptors on cells of the immune system, evidence-based immunomodulatory effects of cannabis *in vivo*, and *in vitro *studies of immune cells (e.g. T cells and macrophages), strongly support the idea that cannabinoids are able to adjust both the function and secretion of cytokines from immune cells. Therefore, cannabinoids seem to be a potential candidate to treat various inflammatory disorders (Croxford and Yamamura, 2005[35]). 

New findings from Cabral et al., (2015[23]) showed drugs like cannabinoids are able to modulate various cytokines, corticosteroids and colony-stimulating factors, which are responsible for the maturation of the stem cells to the competent mature lymphocytes. The thymus and bone marrow are known as the main responsible lymphoid organs capable to convert stem cells to the mature lymphocytes. This maturation of stem cells to the immune cells is very essential to identify and distinguish non self-antigens from foreign antigens instead of self-antigens, therefore reduces autoimmunity (Cabral et al., 2015[23]). Mature lymphocytes leave the bone marrow and thymus, and transfer to the supplementary lymphoid organs (spleen, lymph node, blood, skin, bronchial lymphatic tissue and gut associated lymphatic tissue). As, after the entrance of foreign microbes/antigens, the immune response is triggered by binding cellular elements of immunity (macrophages, dendritic cells, neutrophils, mast cells, eosinophil, basophils, T and B cells) with microbes. Hormones and mediators are mainly responsible for the cellular elements in the bone marrow and thymus. Furthermore, the interactions of cellular elements with antigens/microbes are effected by cannabinoids. Certain effector functions such as cell-mediated immunity, antibody production, signs of allergy, autoimmunity, interleukins and cytokine production linked with mixture of biological active composites like endorphins and anandamide (cannabimimetics) are initiated by antigens (Eisenstein and Meissler, 2015[49]; Kaplan, 2013[80]; Newton, 2001[108]). 

The interaction of an antigen with cytokines or the effector functions provides different entry points at which normal immune homeostasis alters, that makes the drug's effects long and difficult process, as cannabinoids and other drugs conjugate. The immune system has the potential to produce, secrete, carry and metabolize cannabinoids due to the presence of CB1 and CB2 receptors (Bisogno et al., 1997[19]; Cabral and Staab, 2005[25]; Klein et al., 2003[82]; Pestonjamasp and Burstein, 1998[127]). The expression pattern of these receptors is different in each immune system cells. The expressions of these receptors increase in order of; CD4 cells, monocytes, CD8 cells, neutrophils, natural killer cells (NK) and B cells. This tendency of expression has been detected in mouse splenocytes (Bouaboula et al., 1993[20]). The expressions of receptors on immune cells are influenced by cell activation state and immune stimulation (Lee et al., 2001[92]). Multiple studies have shown that exogenous cannabinoids have a vital role in immunosuppression affecting ECS, as a novel therapy for autoimmune and inflammatory disease (Berdyshev, 2000[16]; Cabral and Staab, 2005[25]; Klein et al., 2003[82]; Kumar et al., 2001[87]). 

The escalation of T helper-2 (Th2) cells and lessening Th-1 reactions, also control Th-1/2 balance by the effect of Δ9-THC (Klein et al., 2000[84]; Newton et al., 1994[109]; Yuan et al., 2002[164]; Zhu et al., 2000[166]). Though, the therapeutic effect of Δ9-THC is limited due to its psychoactive effects, somehow, cannabidiols show no psychoactive effects due to their low affinity towards CB1 and CB2 receptors (Munro et al., 1993[105]; Thomas et al., 1998[150]). On the other hands, chronic administration of cannabidiols is tolerable without showing side effects (Consroe et al., 1991[33]). Both *in vivo* and *in vitro* investigations have demonstrated that four main pathways engage in immune suppression of cannabinoids as; initiation of apoptosis, prevention of cell propagation, prevention of mediators, as well as cytokine synthesis and stimulation of T-cells regulatory system (Rieder et al., 2010[133]).

Different studies regarding revealed that anandamide initiate apoptosis in lymphoma U-937 cells, human neuroblastoma CHP-100 cells and mitogen-induced T and B human lymphocytes through a completely dose-dependent manner (Marsicano et al., 2002[100]; Schwarz et al., 1994[139]). It has been illustrated that in murine macrophages and T-cells apoptosis was initiated, through the caspase activity and regulation of BCl_2_ by Δ9-THC (Zhu et al., 1998[167]). Yet, no such evidence of cannabinoids' presentations in induction of apoptosis *in vivo*, as its challenging to determine apoptosis due to the quick and effective clearance through phagocytes (Rieder et al., 2010[133]).

Mice had been injected with Δ9-THC showed reduction in spleen and thymus cellularity, affecting various cells, such as macrophages, T- and B-cells (McKallip et al., 2002[102]). Likewise, the low concentration of Δ9-THC stimulated AnnexinV+ cells, exhibiting early apoptosis, but at higher doses, there was late apoptosis and necrosis due to spleen cell had both AnnexinV and PI positive. Δ9-THC could alter immature lymphocyte instead of active lymphocytes, also the amount of apoptosis reported to be higher in THC treated culture than Δ9-THC and mitogen cultures (McKallip et al., 2002[102]). Accordingly, it is noteworthy that activated lymphocytes can suppress the expression of CB2 receptor and decrease their sensitivity to Δ9-THC. Ingestion of CB2 antagonist blocks Δ9-THC-activated programmed cell death in thymus cells and lymphocytes, so it's observed that Δ9-THC triggers apoptosis via CB2 receptor, while CB1 has no role in this significant effect (McKallip et al., 2002[102]). Cannabidiol induces apoptosis in CD4+ and CD8+ cells, murine thymocytes and EL-4 cells, depending on its concentration and the duration of the experiment. Cannabidiol also initiates apoptosis through the production of reactive oxygen species (ROS) and stimulating caspase-8 and -3 (Lee et al., 2008[91]). 

*In vitro* studies have shown the high doses of Δ9-THC inhibit responses to lipopolysaccharide (LPS), T-cell mitogens and anti-CD3, while lower doses of Δ9-THC trigger T-cells (Klein et al., 1995[83]). The cannabinoids possess a double-phase role in increasing of the proliferation of B-cells in response to Δ9-THC (Derocq et al. 1995[40]), however, another trail indicated a significant decline in response of B-cells to the LPS after cannabinoid therapy (Klein et al., 1995[83]). Cannabidiol improves the production of interleukin-4 (IL-4), IL-10 and Th2-associated cytokines along with decreases in IL-1, IL-12, TNF-α and interferon-gamma cytokines in the peripheral blood mononuclear cells (Weiss et al., 2006[161]). Cannabidiol similarly modifies tissue cyclooxygenase (COX) activity and prostaglandin E-2, while Δ9-THC has the potential to change the critical Th1 immunity to defensive Th2 immunity, though it would be less efficient than cannabidiol (Berdyshev, 2000[16]; Cabral and Pettit, 1998[24]; De Filippis et al., 2008[37]; Munson, 1975[106]; Toguri et al., 2014[152]; Watzl et al., 1991[160]). Δ9-THC showed their positive immunosuppressive effects in *Legionella peumophila* (Lp) infested dendritic cells. Immune suppression was observed in the Lp-dendritic loaded cell pre-treated with Δ9-THC. Inhibition of the maturation markers such as; CD40, CD86, and major histocompatibility complex-II (MHCII) were seen, as Δ9-THC inhibited IL-12p40 production by dendritic cells (Lu et al., 2006[96]).

The T-regulatory cells are resistant to apoptosis distinct from other T-cells induced by Δ9-THC, and may overturn the T-cells that ultimately emit from apoptosis, so further studied are needed (Hegde et al., 2008[72]; Rieder et al., 2010[133]). Several investigations have exposed cannabinoids' receptor ligands can inhibit distribution, cytolysis, mediators proliferation, phagocytosis and antigen expression in the mouse peritoneal macrophages (Cabral and Mishkin, 1989[22]; Carlisle et al., 2002[27]; Gokoh et al., 2005[58]; Lopez-Cepero et al., 1986[95]; Maresz et al., 2005[99]). Additionally, *in vivo* and *in vitro* trials suggested cannabinoids' receptor ligands have the power to suppress natural killer (NK) cells and the cytokine effector functions (Fischer-Stenger et al., 1992[54]; Pross et al., 1992[130]; Wang et al., 1991[157]). Besides above mentioned evidence, some studies also proposed that cannabinoids have pro-inflammatory effects such as endorsing allergic reactions, release of inflammatory cytokines through CB1 receptor in mast cells and enhance B-cells production (Klein et al., 1995[83]; Samson et al., 2003[136]; Small-Howard et al., 2005[146]; Ueda et al., 2007[155]). In optimum concentration, the cells involved in acute and chronic inflammation along with inflammatory reactions can lead to induce programmed cell death in immune cells through cannabinoids. 

### Toxicity of cannabinoids

#### Acute effects 

The short-term cannabinoids' exposure causes very limited toxicity, without eventual death due to direct or instant use of recreational herbal medicine. Statistics indicates that six deaths occurred due to vomiting in upper respiratory tract, pneumonitis and heavily congested lungs, which involved drug intoxication (ethanol, THC, and JWH-018). The most prominent noxious reported effect of cannabis (short-exposure) would be on the cardiovascular system, which shows significant elevation of heart rate and blood pressure falling. Accordance with, individuals with a history of coronary heart diseases, atherosclerosis, cardiomyopathy and other serious cardiovascular diseases are at high risk to cannabinoids toxicity and such subjects (animal model or human) should probably be eliminated from clinical trials of cannabinoids' base studies (Hartman and Huestis, 2013[70]; Nunez and Gurpegui, 2002[111]). Cannabis/cannabinoids short-term effect also involves extreme excitement, which is associated with alcohol and recreational users. Treating individuals with cannabis/cannabinoids show(s) their undesirable side effects along with beneficial properties. Heavy usage of cannabis may damage the intellectual and psychomotor functions being an imperative matter from the public health perspective. The psychomotor function impairment stands from a few hours (h) to 48 h after taking cannabinoids. Individuals compensate for their damages and take less treatments as compared to alcohol tends to boost people to the higher risks and aggressiveness (Cohen et al., 2012[31]; Nunez and Gurpegui, 2002[111]). 

In a study, it was found in road traffic accidents, blood sample analysis indicated 8 % positive for cannabis/cannabinoids, with 10 % fatalities of whom were driving. However, these numbers are controversial, as 22-25 % of cannabis drivers also show the evidence of alcohol consumption as well (Hartman et al., 2015[69]; Sewell et al., 2009[140]). In the same study, cannabis was positive among alcohol positive drivers were as high as 75 %. In this assessment, it was shown the major effects of cannabinoids practice on driving, may increase the damages that are activated by alcohol. In another survey, 1,333 cannabinoids' individuals were examined, users have low accident reports than the general population; those of the highest accident rates were shown to be multiple drug addicts. It is challenging to observe cannabinoids toxicity, as little quantities of cannabinoids release from fatty tissue into the blood stream (Gurney et al., 2014[67]; Hartman and Huestis, 2013[70]; Musshoff et al., 2014[107]; Yeakel and Logan, 2013[163]). First exposure to cannabis with a single dose or overdose in routine individual induces psychological effects such as anxiety, fear, mania and illusion. Cannabinoids may also lead to lifelong psychotic effects, including illusions and the phantasms, which may be misdiagnosed as schizophrenia. Nonetheless, these special effects of cannabinoids are not life-threatening, but cannabis therapy should not be prescribed in these individuals, which may cause serious toxicity when used with other drugs due to drug-drug interaction (D'Souza et al., 2009[36]). The dysphoric reactions of cannabis lead to diverse conditions including; agitation, depersonalization-derealization syndrome, fright and loss of sense (Johns, 2001[79]). It is probably associated with frightening reaction analogous to post-traumatic stress disorder. 

#### Chronic effects 

The extensive uses of cannabinoids have long-term effects on brain functions. Several studies had illustrated that cannabis can affect the attitude, memory, psychomotor performance, sleep, electroencephalogram (EEG), heartbeat, arterial pressure, body temperature and emesis (Gorelick et al., 2012[59]; Hollister, 1986[73]; Lichtman and Martin, 2005[94]). The withdrawal symptoms of cannabis are related to that of alcohol and opioids such as anorexia, muscular tremors, sleeplessness, dysphoria, nervousness, induced reflexes and restlessness. The common signs and symptoms include; weight loss, salivation, loose bowl movements, increased intraocular pressure and nausea (Budney and Hughes, 2006[21]).

Chronic cannabis consumption may also cause psychopathological effects such as suspicious ideation, illusion and hallucination in both psychiatric and non-psychiatric public subjects (Bersani et al., 2002[18]). A cannabis user develops tachycardia with peripheral vasodilatation, which subsequently induces hypotension and reduces body temperature. Moreover, individuals with a history of previous cardiovascular diseases may be provoked by cannabinoids. Nevertheless, even in young men with cardiac infarction, transient ischemic attacks and myocardial ischemia have been observed (Reece, 2009[132]). The constituents of cannabis and cigarette smoke are similar, except nicotine in tobacco causing carcinogenicity of the respiratory tract. The mainstream smoke of cannabis extensively contains benzanthracenes and benzpyrenes, which both are characterized as a human carcinogen (De Oliveira et al., 2008[38]). Cannabis has similar effects to down-regulate humoral and cell-mediated immunity like tobacco. Limited *in vitro* and *in vivo* investigations exhibited that cannabis alters the bactericidal activity of lung macrophages and may reduce respiratory antibacterial defensive system (Tanasescu and Constantinescu, 2010[149]). THC as an eminent cannabinoid binds with an androgen receptor and acts as an antiandrogenic compound. It is proven that long-term exposure to cannabinoids leads to lower sperm counts, sperm motility and abnormal sperm morphology (Park et al., 2004[118]). Chronic use of cannabis enhances prolactin concentration causing galactorrhea and gynecomastia in women and men respectively. 

#### Adverse effects in clinical use

Various classes of cannabinoids such as nabilone, nabiximols and THC are frequently prescribed in clinics. The most common adverse effects of such drugs in recommended doses are sedation, lethargy and dizziness. While, the observed effects were excitation, dysphoria, nervousness, despair, cerebral retardation, recall impairment, obsession and hallucination (Arnold, 2015[7]). It seems recommended doses are mostly prescribed in higher levels as is needed for ill and old patients, therefore, harmful and undesirable consequences could be prohibited from consuming small doses. Generally, nabilone is available in 1 mg, which is 10 times more effective than THC and has a fewer half-life than THC. The plasma removal half-life of parental drugs is estimated to be 2-4 h, whereas those of its metabolites are 20 h, so 84 % of a single dose is removed in 7 days. It is noteworthy that nabilone should be kept away and inaccessible to children and adolescents due to widespread recreational use of it. Different observations have indicated nabilone has slight abuse potential; but in higher doses the euphoric effects are 7 times more severe than THC (Hall, 2015[68]). 

The wide distributions of cannabinoids' receptors in the body have many adverse and beneficial effects. In a systematic review, it has been revealed that cannabinoids have 8, 371 adversarial effects, as 3, 592 were from 8 observational studies and 4, 779 were indicated in 23 randomized controlled trials (Wang et al., 2008[158]). The intensity of adverse effects was 16.5, 16.5 and 15.2 % for respiratory, gastrointestinal and nervous system, respectively in cannabinoids assigned groups, whereas 30 % nervous system conditions mostly stayed in control groups (Wang et al., 2008[158]). In cannabinoids users, 15 deaths were recorded of which 3 belonged to the control group, though the data was non-significant and did not prove that cannabinoids' toxicity was the exact cause. The incidences of severe and non-serious adverse effects reported were higher in cannabinoids users than controls. 

### Cannabinoids unpredicted attribution towards death

Synthetic cannabinoids show typical signs and symptoms as the natural ones (Gunderson et al., 2012[66]; Hoyte et al., 2012[76]; Lemos, 2014[93]). In several case studies, death has been reported as the direct consequences, attributed to synthetic cannabinoids' consumption (Behonick et al., 2014[15]; Saito et al., 2013[135]; Schaefer et al., 2013[138]). Multiple studies illustrated that 4'-methyl-AM-2201 (MAM-2201) is a potent agonist for the cannabinoid receptors, which has been systematically determined in different bio-specimens and no indication of internal or external diagnostic disorder has been reported, but still death occurred due to this drug consumption. The use of herbal blend cannabinoids has initiated seizures. Toxicological analysis of blood samples exhibited existence of five different herbal cannabinoids along with 250 ng/mL amphetamine. Drug intoxication of endo-, phyto-, and synthetic-cannabinoids have attributed to the contributory risk factor of death (Labay et al., 2016[88]). 

Along with the secretion of endo-cannabinoids, physiological cytokines and behavioral toxicity are initiated due to cannabinoids' practice; so, determination of cause and manner of death is essential. Pathological findings, toxicity, initial cause and manner of death in 25 cases are described in Table 1(Tab. 1). The clinical findings and analytical identifications of various cannabinoid compounds were found in the individuals. In 2016, synthetic cannabinoids manufactured by Portuguese Pharmaceutical Company Bail were tested on human volunteers. After progressive completion of phase-I clinical study of animals, the drug was tested on six (6) humans for the first time to ensure safety. Head of Neurology, Pierre-Gilles Edan reported that after the administration, one (1) out of six (6) became brain-dead (coma) and three (3) faced handicap, an irreversible brain damage. In years 2011 to 2015, approximately 20 deaths have been confirmed in the United States due to the extensive use of synthetic cannabinoids (Trecki et al., 2015[154]), while more than 1000 patients were in emergency visits.

### Potential biotechnological approach of cannabinoids

To overcome the toxicity of synthetic cannabinoids, the area of cloning manifested a new scenario regarding biotechnological synthesis of cannabinoids encoding gene THCA synthase (Sirikantaramas et al., 2004[144]). Sirikantaramas et al. (2004[144]) revealed tobacco long-haired roots could generate THCA synthase, which is capable to produce THCA following nourishing of cannabichromenic acid (CBGA). Upon exposure to heat, THCA readily converts to THC (Shoyama et al., 1977[142]). Therefore, such biotechnological advancement leads to produce THA, and then CBGA is easy to manufacture (Mechoulam and Ben-Zvi, 1969[104]; Yagen and Mechoulam, 1969[162]). Hence, this is the need of time that further molecular studies have to be conducted with regard to the biosynthesis of cannabinoids without feeding of some precursors/initiators. Up till now, the cloning and characterization of THCA synthase is possible in the pathway. Previous studies had stated that for the cloning of polyketide synthase, homology-based approach is not efficient (Raharjo et al., 2004[131]). So, metabolomics and proteomics are new branches of omics reported in *C. Sativa*, which can determine various types of metabolites unknown genes involved in the biosynthesis of secondary precursors (Choi et al., 2004[29]). The key point of metabolomics and transcriptomics are particularly applied for the purpose of diagnosing specific disease due to the ability of this advanced technology to accurately detect the reasons, which represent the external changes (Rischer et al., 2006[134]; Tohge et al., 2005[153]; Ziegler et al., 2006[168]). The metabolomics experiments are carried out through either the use of mass spectrometry (MS) or nuclear magnetic resonance (NMR) techniques, which open a way to identify cannabinoid biosynthesis pathway. 

Plant transformation and regeneration hindered genetic engineering in *C. Sativa. *To approach this, *Agrobacterium tumefaciens *is used*,* which potentially transforms *C. Sativa* suspension culture genetically (Feeney and Punja, 2003[52]). Though, the suspension was not capable to synthesize cannabinoids like THCA and cannabidiolic acid (CBDA). Furthermore, genetic engineering was challenging in the culture suspension due to cannabinoids toxicity to *C. Sativa*. The main purpose of biotechnological approaches is to produce transgenic *C. Sativa*, but regeneration of *C. Sativa* is very tough except the formation of somatic embryogenesis from callus (Petri, 1988[128]). Hence, in specific heterologous plants, biomimetic production of cannabinoids would be of interest and applicable. Due to the toxicity of cannabinoids, that would be better to perform such procedures in specific organs like glandular trichomes, which is considered for gene expression. 

In many countries cultivation and possession of *C. Sativa* is illegal. The identification of such drug materials in grabbed samples is critical. The use of anti-THCA monoclonal antibody (MAb) is a landmark for determination of a type of *C. Sativa* (Goto et al., 1994[60]; Tanaka et al., 1996[148]). As, all cannabinoids are cross active to MAb, however, it is just restricted to cannabinoids, *C. Sativa* and its production can be identified from other plants (Sirikantaramas et al., 2007[145]). Therefore, the use of MAb is an indicator of all THC metabolites and it will be a decent screening marker for marihuana consumers (Watanabe et al., 2000[159]).

Another classical determination method for cannabinoids depends on the pollen protein using anti-pollen IgE (Tanaka et al., 1998[147]). The theory behind this method is to distinguish the pollen of *C. Sativa* in the mixture of the other plants' pollen, by the use of deoxyribonucleic acid (DNA) polymorphism of TCHA synthase, which identifies two variants such as *C. Sativa* drug and fiber type in the mixture. This would be possible through the cloning of THCA synthase. A particular polymerase chain reaction (PCR) marker of THCA to distinguish drug type strains has been effectively established (Kojoma et al., 2006[85]; Pacifico et al., 2006[116]).

In area of the drug development, alteration associated with spatial memory has also been explored in the pharmacological field (Egashira et al., 2006[48]). Moreover, study suggested that arachidonic acid cascade plays s vital role in dependency and withdrawal from abused drugs like cannabinoid, opioid and psycho-stimulants (Anggadiredja et al., 2003[6]). Thus, in the endo-cannabinoid system, arachidonic acid cascade assists in the initiation of restoring effect of methamphetamine-primingand signals (Anggadiredja et al., 2003[6]). This shows that endo-cannabinoid activating compounds like cyclooxygenase inhibitors would be working as an antirelapse agent (Anggadiredja et al., 2004[5]). These novel pharmacological phenomena and biotechnological applications would provide better combined results, which developed cannabinoid drugs might be appreciated in future without showing toxicity.

### Regulations about the use of cannabinoids in different countries around the world

**Figure 4:**
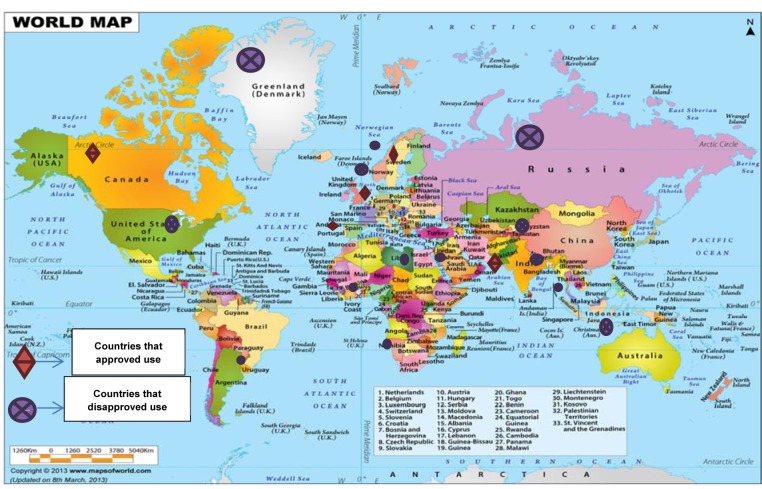
Countries where possession or trade of cannabinoids is considered legal or illegal (www.mapsofworld.com)

The use of cannabinoids (cannabis) has been reported worldwide. A survey conducted in 2009, provided a rough estimate of users according to region and sub-region. The utilization of cannabinoids have been legalized in several countries including; Austria, Germany, Canada, Finland, Italy, Netherlands, Israel, and many others, whereas possession or trade of these compounds is considered illegal in elsewhere (Parry and Myers, 2014[119]). However, it has not been approved by the United State Food and Drug Administration (FDA). For instance, the users of cannabis in Australia are facing some penalties such as cautioning, heavy fines and imprisonment; depending on the state, age of the individual user and the amount of possession (Campbell, 2001[26]; Barratt et al., 2013[12]). Figure 4(Fig. 4) provides a summary of some countries that possession or trade of these compounds is considered legal or illegal.

## Conclusion

It is concluded that specific synthetic cannabinoids possess poor responses towards CB1 or CB2 receptors, while some synthetic members like Δ9-THC exhibit high affinity to these receptors. That might be due to the fact that cannabinoids initiate immune modulatory effects without any psychological signs. They play potential and effective roles in certain autoimmune diseases. Only few studies are available regarding immunosuppressive properties of cannabinoids, still further pinpoint and accurate investigations are required. Cannabinoids have shown direct or indirect association with mortality, however, their usage should be defined during post-mortem of various cases, when specified by exploratory and case history. It is clear that the extreme use of cannabis and cannabinoids conveys risks, both to the public and the habitual users. Therapeutic applications of such compounds need careful attention and critical scientific point of view. Studying the effects of natural cannabinoids and more importantly the synthetic types (due to their higher toxicity) on the receptors could help clinicians to govern the adverse incidence and expand the pharmacological and medicinal applications associated with them. 

Challenges like the safety profile of these compounds, complete understanding of their biosynthetic pathways and the activities of the enzymes and receptors involved in their metabolism throughout the body remain to be resolved. In order to use the biotechnological techniques for cannabinoid production is possible to overcome the toxicity. In this manner, the application of biotechnological techniques like cloning and monoclonal antibody practices to improve the synthesis of cannabinoids like the encoding gene for THCA synthase might help to overcome the toxicity of these compounds. The combination of pharmacological and biotechnological applications might lead to the development of some cannabinoid drugs in future without showing toxicity. Further experimental studies and case reports are needed for the toxicologists and forensic pathologists to determine the specific cause of death from the case history, biopsy, clinical findings and post mortem lesions. 

## Acknowledgements

This article is the outcome of an in-house financially non-supported study. Authors wish to thank Iran National Science Foundation (INSF).

## Author contributions

All authors have directly participated in the planning or drafting of the manuscript and read and approved the final version.

## Conflict of interest

The authors declare no conflict of interest.

## Figures and Tables

**Table 1:**
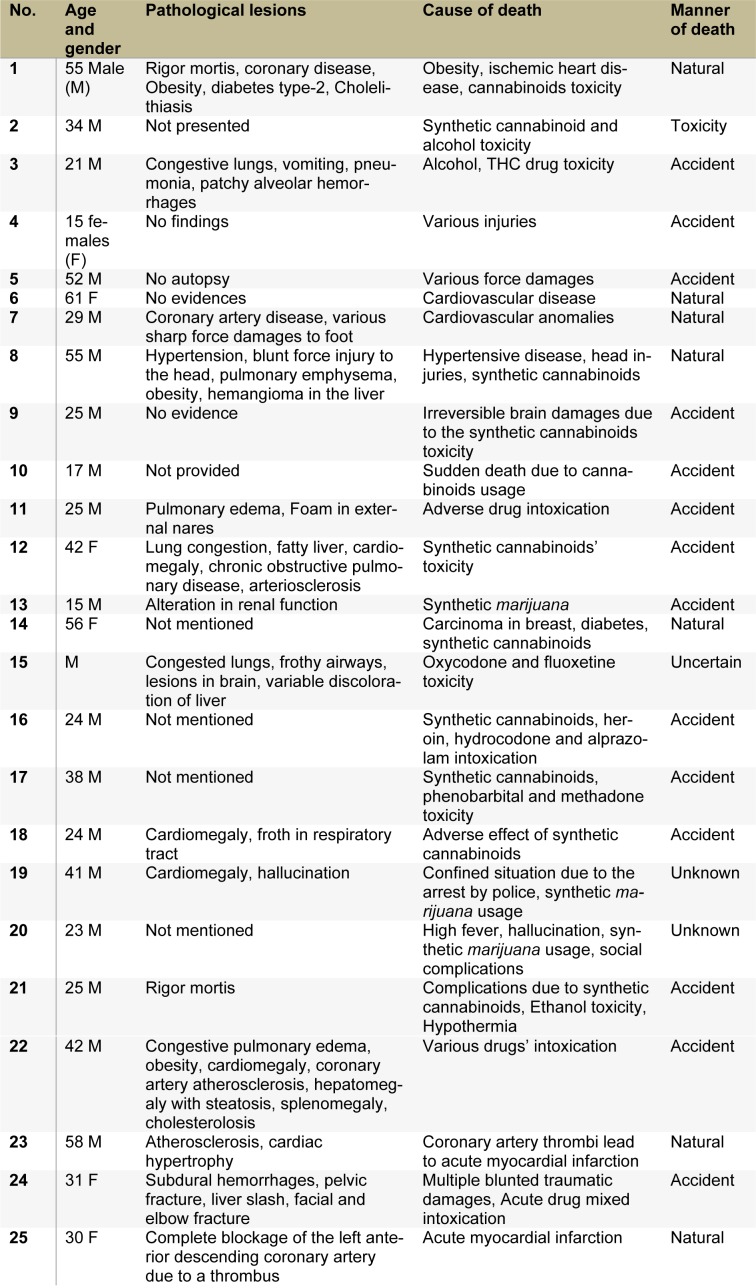
Pathological findings, the cause and manner of death, a comparison of cannabinoids toxicity with other drug intoxication
